# Structural insights into the modulation Of SOD1 aggregation By a fungal metabolite Phialomustin-B: Therapeutic potential in ALS

**DOI:** 10.1371/journal.pone.0298196

**Published:** 2024-03-06

**Authors:** Sruthi Unni, Padmini Kommu, Snehal Aouti, Yedukondalu Nalli, M. M. Srinivas Bharath, Asif Ali, Balasundaram Padmanabhan

**Affiliations:** 1 Department of Biophysics, National Institute of Mental Health and Neurosciences, Bengaluru, India; 2 CSIR-Indian Institute of Integrative Medicine, Natural Product Division, Jammu, India; 3 Department of Clinical Psychopharmacology and Neurotoxicology, National Institute of Mental Health and Neurosciences, Bengaluru, India; 4 Medicinal & Process Chemistry Division, CSIR-Central Drug Research Institute, Jankipuram Extension, Lucknow, India; Indian Institute of Technology Guwahati, INDIA

## Abstract

Amyotrophic lateral sclerosis (ALS) is a fatal human motor neuron disease leading to muscle atrophy and paralysis. Mutations in superoxide dismutase 1 (SOD1) are associated with familial ALS (fALS). The SOD1 mutants in ALS have a toxic-gain of function by destabilizing the functional SOD1 homodimer, consequently inducing fibril-like aggregation with a cytotoxic non-native trimer intermediate. Therefore, reducing SOD1 oligomerization *via* chemical modulators is an optimal therapy in ALS. Here, we report the discovery of Phialomustin-B, an unsaturated secondary metabolite from the endophytic fungus *Phialophora mustea*, as a modulator of SOD1 aggregation. The crystal structure of the SOD1-Phialomustin complex refined to 1.90 Å resolution demonstrated for the first time that the ligand binds to the dimer interface and the lateral region near the electrostatic loop. The aggregation analyses of SOD1^WT^ and the disease mutant SOD1^A4V^ revealed that Phialomustin-B reduces cytotoxic trimerization. We propose that Phialomustin-B is a potent lead molecule with therapeutic potential in fALS.

## Introduction

Amyotrophic lateral sclerosis (ALS) is a fatal neurodegenerative disorder wherein a severe progressive loss of motor neurons leads to paralysis and gradual death [1,2]. Motor neurons in ALS patients revealed the presence of prion-like protein aggregates, a hallmark in ALS pathophysiology. Single residue mutations in the antioxidant enzyme superoxide dismutase 1 (SOD1) are reported in familial ALS (fALS) patients throughout the world [3,4]. The conformational changes induced in the SOD1 structure by these pathogenic mutations lead to its oligomerization and progressive fibrillation or fibrillization. The cytotoxic aggregation has been reported in self-seeded and cross-seeded complexes involving other proteins such as TDP-43 and FUS [5,6]. The proteinopathy in fALS is caused by a toxic gain of function of SOD1 [7,8], causing neurotoxicity and iron metabolism dysregulation [9,10]. The native structure of SOD1 is a homodimer of 32kDa size. The intramolecular disulfide bond and Cu-Zn metal coordination impart higher structural stability and keep the dimer intact. This dimeric structure dissociates into monomers, followed by oligomerization under disease conditions. Also, various studies showed a non-native trimeric intermediate formation with a toxic gain of function called the toxic trimer species. The trimeric species are formed by the aggregation of partially unfolded monomers, which have segregated from the native dimer [11–13]. Preventing the formation of lethal trimer species is a potential strategy for preventing neurodegeneration in ALS [11].

The complicated pathogenesis of ALS has limited the development of therapeutic interventions. Although ALS-causative genetic mutations have been discovered, there is still no definitive test to detect the disease. The current diagnosis of the disease depends on excluding other progressive upper and lower neuronal motor dysfunctions [1,14]. Because ALS is an immensely time-dependent disease, the delay in diagnosis progressively limits the motor capabilities of ALS patients. Therefore, current treatment practices include symptom management through metabolic assessment tests for respiratory care, dysphagia, cognitive impairment, and overall palliative care [1]. Although pharmacotherapeutic drugs are available for ALS management, they have certain limitations. Riluzole, a glutamate-release inhibitor, was approved by the USFDA as a prescription drug to manage the progression of ALS [15]. It only prolongs the survival duration by approximately three months [16]. The other USFDA-approved drug, Edaravone, has antioxidant properties. Still, its exact mode of action is unknown in ALS, and its access is limited due to high costs and lack of oral administration. There are also symptom-specific drugs prescribed to ALS patients, such as quinine and baclofen for muscle cramps, NSAID for pain, lorazepam, diazepam, alprazolam, or buspirone for anxiety [17,18]. However, their effect is limited till targeting the symptom. Combination therapy of acamprosate and baclofen (PXT864) has been shown to prevent glutamate excitotoxicity and TDP-43 inclusions in ALS models [19]. Hence, extensive studies aimed to discover novel drugs that delay disease progression and prevent proteinopathy and aggregate formation. It has been proposed that small molecules that stabilize the SOD1 dimer or inhibit tryptophan (W32) oxidation could prevent aggregation. Small molecules that target the surface exposed W32 potentially inhibit oxidation and prevent the fibrillation [20–23]. Another strategy employs small molecules such as cisplatin [24], ebselen, and ebsulphur [25,26] that covalently bind to the free cysteine, C111 and zip lock the SOD1 dimer. However, no compounds have been discovered so far that a ligand binds to SOD1 other than tryptophan (W32) and cysteine (C111) sites.

We have previously reported the isolation of four distinct azaphilone-derived molecules, Phialomustin A-D, from an endophytic fungus, *Phialophora mustea*, isolated from *Crocus sativus*. These isolated molecules exhibited antimicrobial and cytotoxic potential [27]. In the current study, using structure-based screening, we have discovered a potent unsaturated secondary metabolite designated as Phialomustin-B (PB, hereafter) from *Phialophora mustea* as a modulator of SOD1. PB is a linear unsaturated fatty acid chain designated as (S, 2E, 4E)-4, 6-dimethyldeca-2, 4-dienoic acid. Intriguingly, in the present crystal of the SOD1-PB complex refined to 1.90Å resolution, the PB ligands bind to SOD1 at two new binding sites: close to the dimer interface and the lateral region, which is close to the electrostatic loop. Moreover, biochemical studies revealed that PB could reduce the formation of time-dependent toxic trimers, and hence, PB may be a potential lead molecule for library development for inhibiting SOD1 toxic trimer-inducing aggregation.

## Results

### Phialomustin-B (PB), a fungal derivative molecule from *Phialophora mustea*

We have recently reported the rational drug discovery of a SOD1 inhibitor by computational, biochemical, and crystal structure studies [20]. When we further analyzed the weak binding compounds by co-crystal studies, we observed an apparent electron density for one of the SOD1-ligand complexes. Although the ligand was not found in this complex, we observed electron density corresponding to an unknown molecule (S1 Fig). Unexpectedly, this unknown linear molecule (maybe a solvent molecule used in the ligand isolation) is found near the dimer interface region based on the electron density. Subsequently, we have taken these unexpected findings as a lead to screen an in-house bioactive library of 30 compounds from *Phialophora mustea* and *Piper mullesua*. We obtained a SOD1 complex with a fungal metabolite, Phialomustin-B (PB), from the exhaustive co-crystallization screening. PB is a distinct unsaturated fatty acid isolated from an endophytic fungal strain, *Phialophora mustea*. Endophytic funguses are one of the richest sources of secondary metabolites with various therapeutic efficacies. Endophytes’ proficiency in producing diverse secondary metabolites was the inspiration to explore the isolation of the bioactive metabolite from an endophytic fungus, *Phialophora mustea*, isolated from corms of the botanical plant *Crocus sativus* [27].

### Crystal structure of the complex SOD1-PB

The X-ray diffraction data for a co-crystal of SOD1^WT^ in complex with the ligand, PB, was collected on the ID30A beamline, ESRF, Grenoble, France. The crystal diffracted up to 1.90Å resolution. The crystal belongs to the hexagonal space group, P6_3_22, with five SOD1 dimers in the asymmetric unit. The structure was solved by molecular replacement using the apo-form of hSOD1 as a model (PDB ID: 5YTO). The final refined structure of the SOD1 complex in the asymmetric unit contains 11074 protein atoms, nine Zn^2+^ ions, four PB ligands, five glycerol molecules, and 1208 water molecules, with a final R_work_ of 19.8% and a R_free_ of 23.0% at 1.9 Å resolution The R_work_ and R_free_ are used to judge the quality of the final structure model. The R-factor (also called the residual factor) is a measure of agreement between the crystallographic model and the experimental X-ray diffraction data. The Free R-factor (R_free_) is a quality control parameter to assess the accuracy of the final structure model. The X-ray data collection, scaling, and refinement statistics of the SOD1-PB complex are summarized in Table 1.

**Table 1:** Data collection and final refinement parameters for the crystal structure of SOD1^WT^ in complex with a fungal isolate compound, PB.

Data collection parameters	SOD1^WT^—PB
Space group	P6_3_22
Cell dimensions a b c (Å)	242.44, 242.44, 144.49; γ = 120º
Wavelength (Å)	0.967
Resolution (Å)^#^	50.0–1.9 (1.93–1.90)
Unique reflections	195483 (9652)
R_meas_ ^†^	0.11 (1.64)
⟨I/σ⟩	19.9 (1.24)
Completeness (%)	100 (100)
Redundancy	9.6 (8.2)
CC_1/2_^##^	0.948 (0.719)
**Refinement**	
Resolution (Å)	38.6–1.90
No. of reflections	194865
^ǂ^R_work_ / ^+^R_free_ (%)	19.8/23.0
Total no. of atoms	12385
Protein atoms	11074
Water molecules	1208
Ligand molecules	4
Zn-ions	9
Glycerol molecules	5
Average B-factor (Å^2^)	27.6
RMSD Bonds (Å)	0.008
RMSD Angles (°)	1.10

^#^Numbers in parentheses are values in the highest resolution shell.

^†^R_merge_ = ∑_hkl_ ∑_i_|I_i_ (hkl) − 〈I(hkl)〉|/(∑_hkl_ ∑_i_ I_i_ (hkl), where *I*_*i*_*(hkl)* is the intensity of the *i*^*th*^ measurement and ⟨*I(hkl)*⟩ is the mean intensity for that reflection.

^ǂ^R_work_ = ∑_hkl_||F_obs_| − |F_calc_||/∑_hkl_|F_obs_|, where |*F*_*obs*_| and |*F*_*calc*_| are the observed and calculated structure-factor amplitudes, respectively.

^+^*R*_*free*_ was calculated with 5.0% of reflections in the test set.

^##^Values correspond to the highest resolution shell.

**Fig 1:**
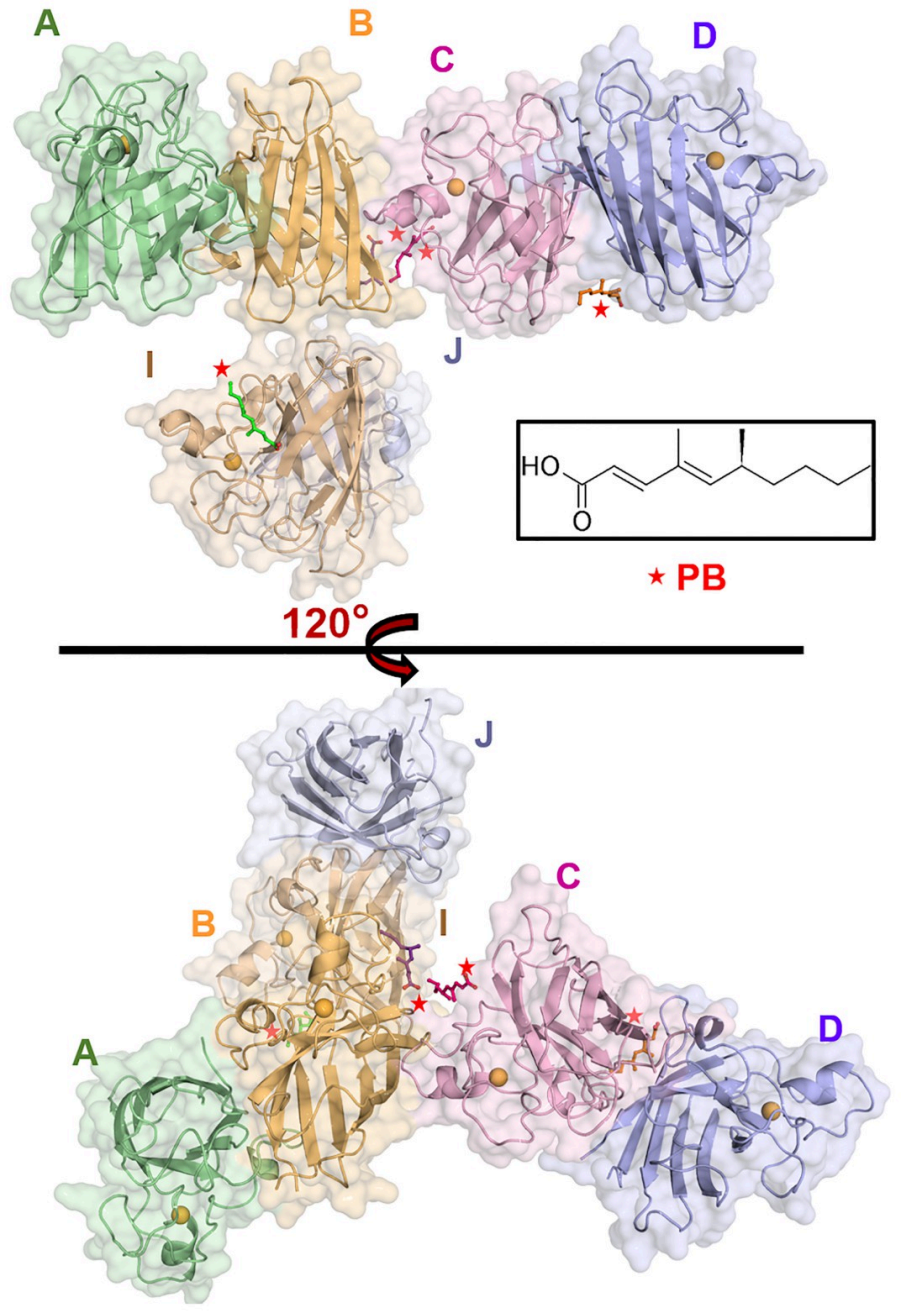
Crystal structure of human SOD1 complex with the ligand Phialomustin-B (PB). The surface/cartoon model of the assembly of SOD1-PB dimers in the space group P6_3_22 shows binding from PB (shown in the sticks) at four positions, one of them at the SOD1 dimer interface. The two-dimensional structure of PB is shown inset. A red star indicates the position of PB.

**Fig 2:**
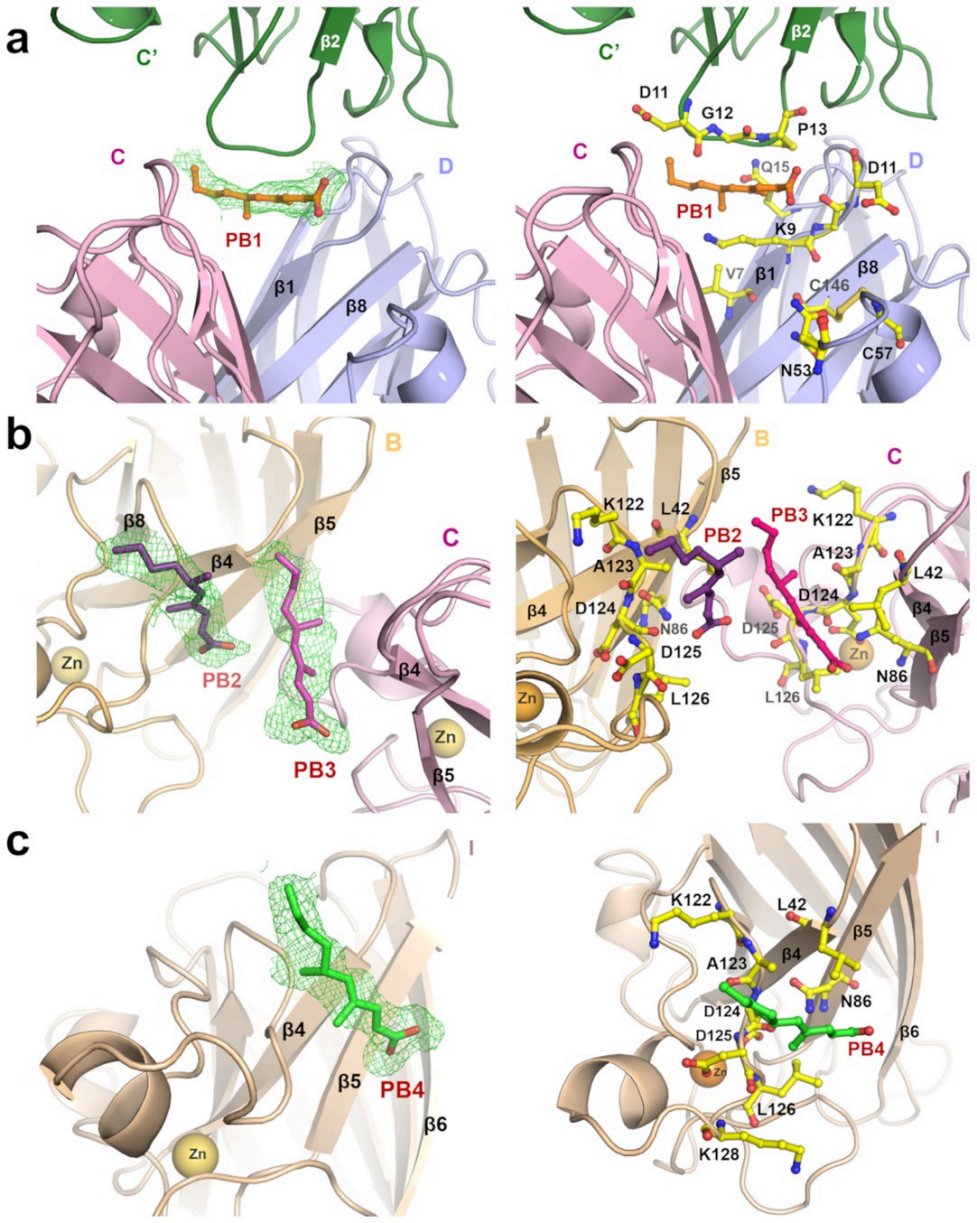
Detailed analysis of the four binding sites of PB on SOD1. The polder density map and the intermolecular interactions of PB1 (shown as orange sticks) on the dimer interface of chains C (shown as a pink cartoon) and D (shown as a blue cartoon) are shown in **(a)**; right and left panels, respectively. The polder map for the other poses on the lateral region of chains B (shown as a golden yellow cartoon), C, and I (shown as a wheat brown cartoon) are shown in **(b)** and **(c**) (left panels). The intermolecular interactions of PB2 (purple sticks), PB3 (pink sticks), and PB4 (green sticks) are shown in **(b)** and **(c)** (right panels). The Polder maps are contoured at the 3σ level for PB1 and PB2, 3.3σ level for PB3, and 3.5σ level for PB4. The maps are shown as green mesh. The binding site residues are shown as yellow sticks.

Four PB ligand molecules are bound to the SOD1 proteins in the asymmetric unit (Fig 1). Intriguingly, all these ligand molecules bind at new binding sites of hSOD1. Here, we report the first co-crystal structure of the SOD1 in a complex with a ligand, which shows binding at the new sites. The first site of ligand (PB1) binding is found near the dimer interface of the CD dimer and near the intra-disulfide bond regions in both the chains (Fig 2a), while the other three ligands (PB2-4) bind in the lateral region of SOD1 molecules in chains B, C and I (Fig 2b and 2c). The residues involved in the intermolecular interactions are shown in Table 2. The ligand-binding site at the dimer interface is formed by the C-term part of the β1 strand, a loop connecting β1 & β2 strands, the β8-strand and the N-term part of the Zn-loop in chains C and D **(**Fig 2a). The ligand, PB1, is bound close to chain D and the loop connecting the β1 & β2 strands of the symmetry chain C (C’) (Fig 2a). The compound is wedged between the K9 and Q15 side chains of the chain D and D11-P13 residues of chain C’. The ligand is supported by hydrophobic interactions from the carbon side chain of K9 of chain D and P13 of chain C’.

**Table 2:** List of interactions between PB molecules and SOD1 residues as seen in the crystal structure.

Binding Site in SOD1	Ligand molecule	Interacting residues
Site 1: Dimer Interface β1, β2, β8 β-strands of SOD1	PB1	Chain D: K9, G10, D11, and Q15 Chain C’: D11, G12, and P13
Site 2: Electrostatic Loop And β-strands β4 and β5	PB2	Chain B: L42, K122, A123, D124, and D125
	PB3	Chain C: L42, N86, A123, and L126
	PB4	Chain I: L42, N86, A123, and L126

The binding mode of the other three ligands, PB2-4, at the lateral site of SOD1 molecules (chains B, C, and I) in the asymmetric unit is almost similar. The ligands lie on the surface produced by the electrostatic loop, and the β-strands β4 and β5 (Fig 2b and 2c). For the compounds PB3 and PB4, which bind to the chains C and I, respectively, the carboxylate moiety makes with N86 of respective chains. The aliphatic part of the ligands contributes to hydrophobic interactions with L42, A123, and L126. The other ligand, PB2 binds to chain B similarly to the PB3 and PB4 binding; however, the carbonyl moiety of PB2 makes a hydrogen bond with the carbonyl group D124 instead of N86 (Fig 2b).

### Comparison of the ligand PB bindings in the lateral site region

**Fig 3:**
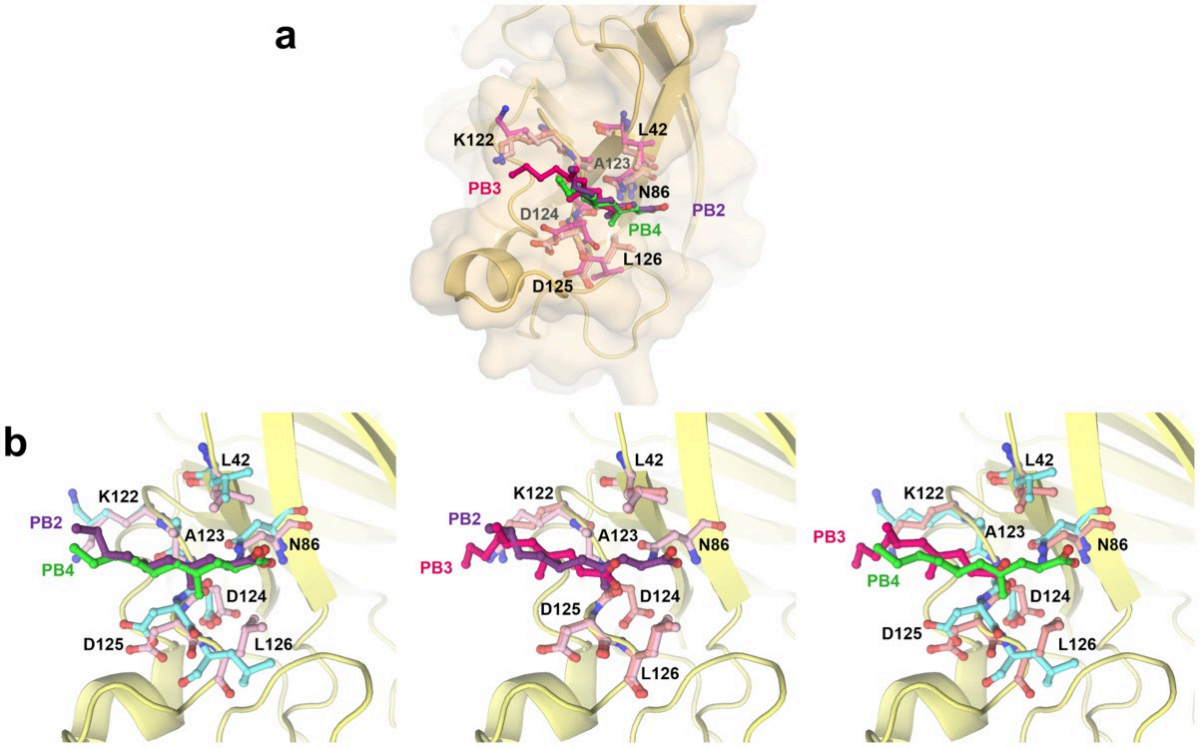
Structural comparison of the binding poses of PB. **(a)** Superposition of the binding poses of PB2 (purple sticks), PB3 (pink sticks), and PB4 (green sticks) at the lateral binding site. **(b)** Comparison of the orientation of PB3-PB4 (left panel), PB2-PB3 (center panel), and PB2-PB4 (right panel) and the binding site residues for each of the chains. The binding site residues of chains B, C, and I are shown as light purple, light pink, and cyan sticks.

The PB ligand is bound at the lateral site region of three SOD1 chains in the asymmetric unit (chains B, C, and I) (Fig 3a). On superposition of three ligands onto each other, it is revealed that PB anchors to the binding site through ionic interactions with the residues N86 (chain C and I) (Fig 3b) and the backbone carbonyl group at D124 residue (chain B) (Fig 3c and 3d). Further, hydrophobic interactions are observed between the aliphatic body of PB with the leucine and alanine residues. The overall mode of intermolecular interactions at the lateral site is nearly similar.

### Evaluation of the ligand, PB binding with SOD1^WT^ through microscale thermophoresis assay

**Fig 4:**
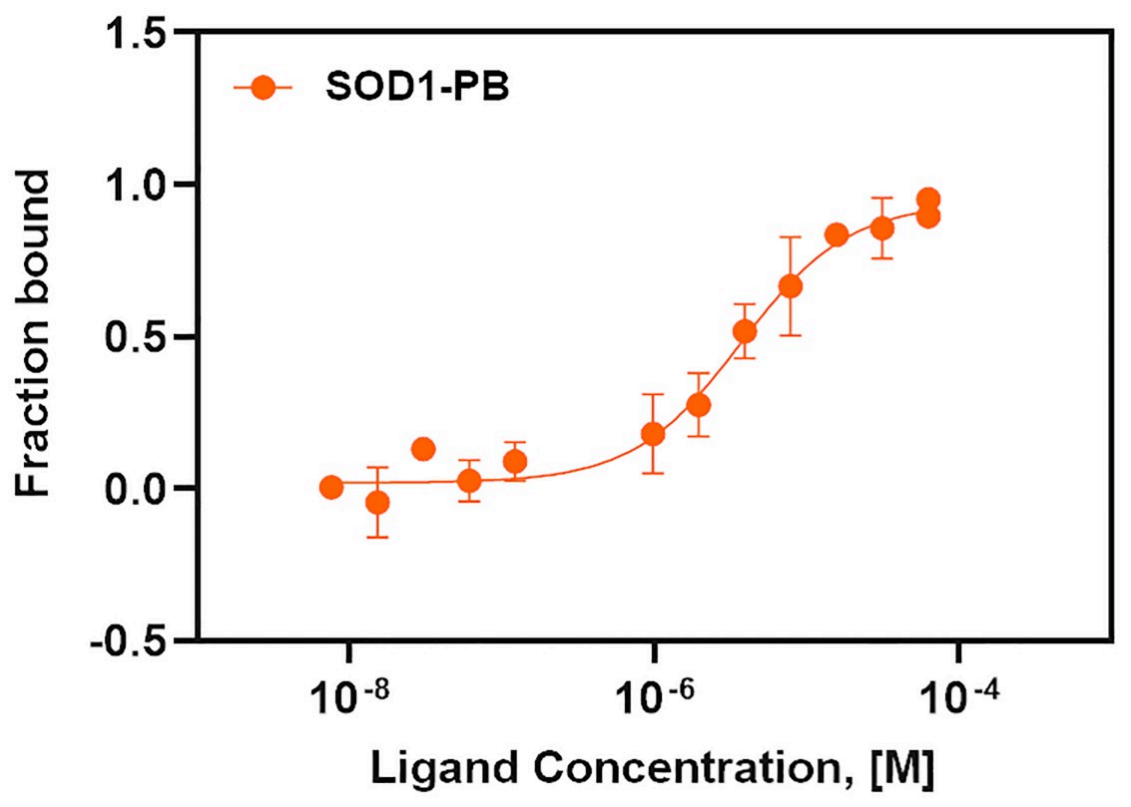
MST interaction analysis of fluorescently labeled SOD1^WT^ against PB. The binding of PB to fluorescently Cys-labeled SOD1^WT^ is quantified in PBS buffer. The concentration of PB varied from 30.5 nM to 250 μM, while the protein SOD1^WT^ was kept constant at 25 nM. MST experiments were conducted at 20% LED excitation and medium MST power at 25 °C. The resulting dose-response curves were fitted, and a binding affinity of K_d_ = 3.7 ± 1.2 μM (orange circles) was calculated. Error bars indicate the standard error of the mean, SEM (n = 3).

To evaluate the binding efficiency of the ligand PB with SOD1^WT^, a quantitative binding assay by microscale thermophoresis (MST) was performed. SOD1^WT^ was labeled at its cysteine residues to ensure non-interference with lysine-containing binding sites of PB (K9, K122, and K128). The cysteine label targeted free cysteines lying away from the ligand-binding site region. The fluorescently labeled SOD1^WT^ and 16 different concentrations of PB from 250 μM to 30.5 nM were prepared to evaluate the binding interactions between SOD1^WT^ and PB, as discussed in the methodology. The MST traces exhibited a dose-dependent shift in their magnitudes. The binding affinity was calculated based on the directional movement of molecules along a temperature gradient and the thermophoretic properties of the protein-ligand molecules. The binding affinity of the compound PB with SOD1^WT^ was determined at 3.7 ± 1.2 μM (Fig 4).

**Fig 5:**
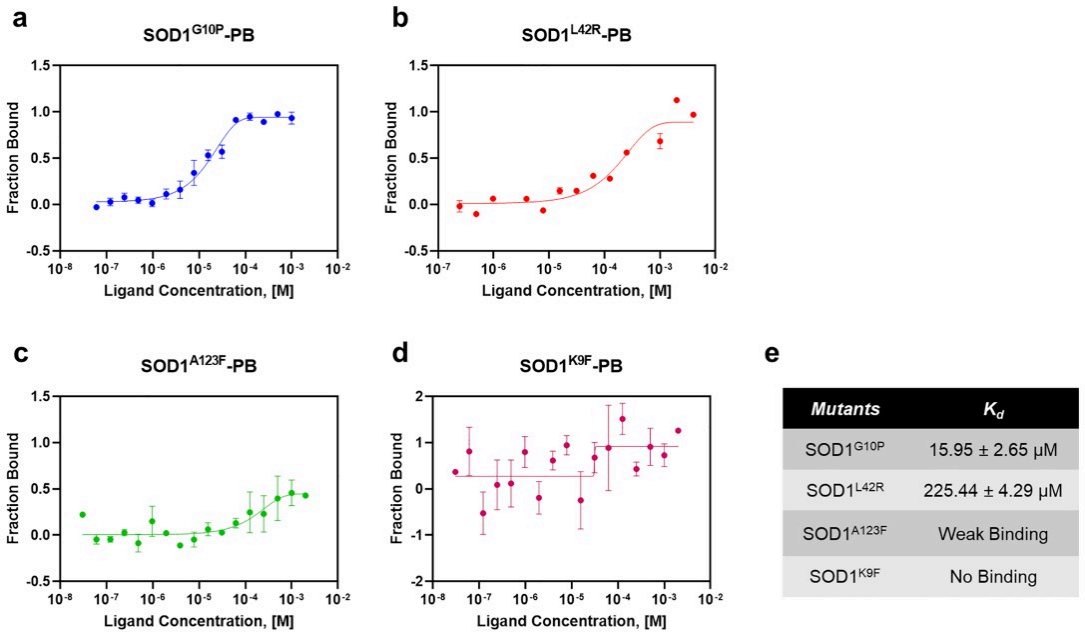
MST interaction analysis of fluorescently labeled SOD1 mutants against PB. The binding of PB to fluorescently Cys-labeled SOD1- variants is quantified in PBS buffer. The concentration of PB varied from 30.5 nM to 250 μM, while the respective mutants were kept constant at 25 nM. MST experiments were conducted at 20% LED excitation and medium MST power at 25 °C. The resulting dose-response curves were fitted and yielded the binding affinity of **(a)** 15.95 ± 2.65 μM for SOD1^G10P^, **(b)** 225.44 ± 4.29 μM for SOD1^L42R^, **(c)** weak-binding for SOD1^A123F^, and **(d)** no binding for SOD1^K9F^. Error bars indicate the standard error of the mean, SEM (n = 3).

To further validate the binding of PB to SOD1, site-directed mutagenesis was performed for the interacting residues at the binding sites of PB. The point mutations generated on the SOD1 protein are K9F, G10P, L42R, and A123F. The MST assay was carried out on these mutants against PB (Fig 5). The dose-response curve with the highest signal-to-noise ratio, high response amplitude, and lowest error rate was selected for measuring the binding affinity (K_d_) value. The K_d_ values for mutants were significantly higher than the wild-type protein (Fig 5), confirming the ligand binding specificity. By comparing the binding efficiencies, K9F significantly affected the binding affinity while mutating at G10 caused the least effect on ligand binding. Binding at the electrostatic loop is affected majorly by mutating residues at A123 to phenylalanine and L42 to arginine. The binding affinities for the compound PB with the mutants SOD1^G10P^ and SOD1^L42R^ are 15.95 ± 2.65 μM and 225.44 ± 4.29 μM. The mutant SOD1^A123F^ shows very weak binding, while SOD1^K9F^ shows no binding with the ligand. Taken together, the binding affinities of these mutants confirm the ligand bindings at these sites of SOD1 are specific.

### PB treatment reduces the formation of toxic trimer species in metallated SOD1^WT^ and an ALS disease mutant, SOD1^A4V^

**Fig 6:**
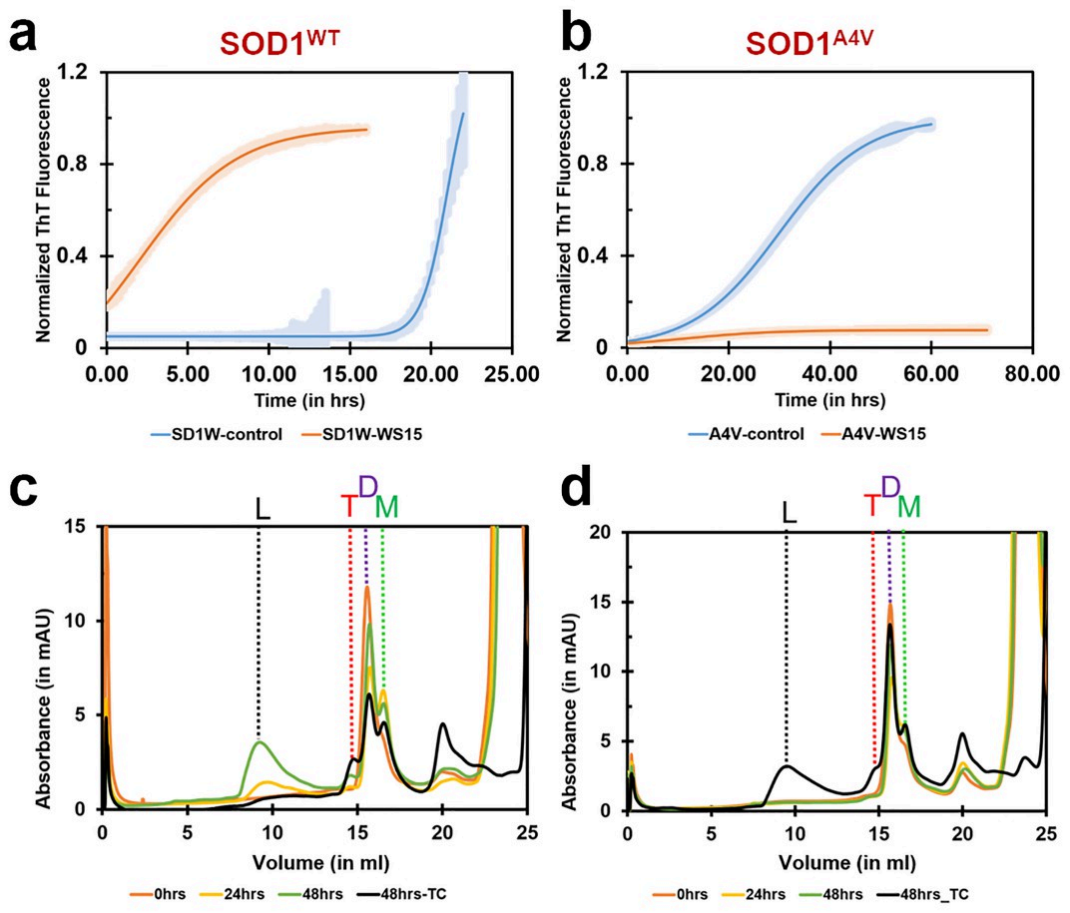
Aggregation studies of metallated and reduced SOD1^WT^ and SOD1^A4V^ on treatment with PB. ThT fluorescence was monitored during the co-incubation of metallated SOD1^WT^ (50 μM) **(a)** or SOD1^A4V^ (50 μM) **(b)** with PB at 1:30 molar ratio at 37 °C with continuous shaking, under reduced conditions. The values were normalized to the maximal ThT intensity elicited by SOD1^WT^ or SOD1^A4V^ and fitted to a Boltzmann sigmoidal equation. The control and PB-treated species were compared to analyse the effects of PB on the aggregation of SOD1^WT^ and SOD1^A4V^. Error bars indicate the standard deviation. Analytical SEC of metallated SOD1^WT^ (50 μM) **(c)** and SOD1^A4V^ (50 μM) **(d)** with PB were analyzed under reducing conditions before and after 24 hrs and 48 hrs incubation at 37ºC. The first peak eluting at the volume was denoted as oligomerized SOD1^WT^/SOD1^A4V^ (L). The second, third, and fourth peaks were calculated as trimer (T), dimer (D), and monomer (M) species of SOD1^WT^/SOD1^A4V^ running at 14.5 ml, 15.7 ml, and 16.5 ml, respectively. Size-exclusion chromatogram shows how PB binding to SOD1^WT^/SOD1^A4V^ promotes shifting towards the formation of large aggregates (L) rather than toxic trimer (T) formation in comparison to the control (48hrs-TC, TC represents the Test Control). The monomer (M), dimer (D), trimer (T), and large aggregate (L) species are shown as green, purple, red, and black dotted lines, respectively. All the experiments were performed for n = 3 biological replicates.

Following the co-crystal structure elucidation of SOD1 in complex with PB, we wanted to identify the effect of PB on the aggregation of SOD1^WT^ and its disease mutant, SOD1^A4V^. A comprehensive analysis of the anti-aggregation propensity of PB was performed using a ThT-based fluorescence assay and analytical size-exclusion based chromatography (SEC) assay. We used Thioflavin-T, a β-sheet formation reporter, to assess the aggregation of SOD1. Time lag (t_lag_) is a kinetic parameter used to evaluate the lag phase of fibrillation. Fibril elongation was used to evaluate the rate of β sheet formation [23]. Analysis of the ThT results revealed that PB initiated a faster t_lag_ for both the metallated proteins (SOD1^WT^ and SOD^A4V^) in the presence of the ligand PB (Fig 6a and 6b; Table 3). Although the lag time reduces considerably in the case of SOD1^WT^, the elongation phase shows significantly slower aggregation on treatment with PB. Similar effects on lag time are also observed for SOD1^A4V.^ Additionally, the treatment of PB revealed a drastic reduction in the ThT signal of SOD1^A4V^ aggregation. The results suggest that earlier but slower aggregate formations tend to occur both in SOD1^WT^ and SOD1^A4V^ on treatment with PB. Moreover, ThT-based aggregation assays were also performed for the PB site-specific mutants, SOD1^A123F^, SOD1^K9F^, SOD1^L42R^, and SOD1^G10P^ (S4a, S4c, S4e and S4g Fig). As expected based on the abovementioned MST results, the aggregation analysis is inconclusive as these mutants, which correspond to the ligand binding residues, cause a loss of protein-ligand interactions.

**Table 3:** The kinetic parameters for metallated and de-metallated conditions of SOD1 in the presence or absence of PB.

S.No.	Condition	Protein	k_app_ (h^-1^)	t_50_ (h)	t_lag_ (h)
**1.**	Metallated	SOD1^WT^	0.063	11.05	11.04
**2.**		SOD1^WT^-PB	0.233	2.976	2.974
**3.**		SOD1^A4V^	0.028	24.93	24.91
**4.**		SOD1^A4V^-PB	0.07	10.45	10.44
**5.**	De-metallated	SOD1^WT^	0.054	12.81	12.79
**6.**		SOD1^WT^-PB	0.374	1.851	1.84
**7.**		SOD1^A4V^	0.13	5.32	5.31
**8.**		SOD1^A4V^-PB	0.05	13.57	13.55

Time-resolved analytical size-exclusion chromatography was also carried out to analyse the change in the conformational arrangements of SOD1^WT^ and SOD1^A4V^ on treatment with PB under controlled *in-vitro* aggregating conditions. Based on the reported studies, PB’s anti-aggregation propensity was efficiently performed on metallated and de-metallated species of SOD1^WT^ and SOD1^A4V^ under reducing conditions [28–34]. The analytical size-exclusion based chromatographic aggregation assays were performed for the SOD1^WT^ and SOD1^A4V^ species in the presence of positive controls (S2 Fig). The elution volumes of the dimer, trimer and monomer populations were 15.7 ml, 14.5 ml, and 16.5 ml, respectively. The dimer, trimer, and monomer populations were validated using a calibration curve (not shown). The monomeric A4V is known to trimerize with other monomeric A4V and form into non-native toxic trimer species over time [35]. A similar time-dependent formation of trimeric A4V is observed in the assayed setups, but the concentration of monomeric species remains constant over time. In the absence of PB, the concentration of dimer and trimer species experience a time-dependent increase and decrease, respectively. (S2c and S2d Fig). The results for analytical size-exclusion chromatography revealed that treatment of SOD1^WT^ with PB may induce the formation of higher aggregates in a time-dependent manner (Fig 6c). This oligomer formation (eluting at 9.2 ml) was accompanied by time-dependent alleviation of toxic trimer population and marginally less monomer formation. An increase in the dimer formation was noticed at 48 hours compared to 24 hours. We observed a time-dependent increase in the ratio of the dimer to the monomer.

Over the course of previous studies, researchers have been able to derive the formation of trimer species of SOD1 that have been proven to be toxic to motor neuron-like cells [36]. The oligomer formation (eluting at 9.2 ml) seen in the SEC aggregation assay was accompanied by time-dependent alleviation of toxic trimer population and marginally less monomer formation. An increase in the dimer formation was noticed at 48 hours compared to 24 hours. We observed a time-dependent increase in the ratio of the dimer to the monomer. Previous studies also revealed that formation of larger SOD1 aggregates form as a protective mechanism in motor neuron-like cells [12]. Therefore, the skewing of balance between the formation of trimers and large aggregates decides the therapeutic potential of the molecule.

Investigation of the anti-aggregation propensity of PB on SOD1^A4V^ revealed that under reducing conditions, treatment of PB at a ratio of 1:30 visibly reduced the formation of toxic trimer species (Fig 6d). To validate the dose-dependent changes in the formation of populations, an increased dose of PB (60X) was introduced into the assay setup. The treatment with a higher concentration of PB increases aggregate formation of SOD1^A4V^. It was observed that the formation of SOD1^A4V^ monomer species remains unaffected by the treatment of PB. Although the increased ligand concentration introduced a dose-dependent formation of higher aggregates (at an elution volume of 9 ml), trimer populations were significantly reduced (S3a Fig). Moreover, as observed in the reported studies [22], a ligand control was also run without the proteins SOD1^WT^ or SOD1^A4V^ to verify that the aggregate formation occurs in the presence of protein and ligand (not shown). As expected, the ligand control revealed no aggregate formation. Hence, the oligomer formations occurred due to the interaction of PB with SOD1^WT^ or SOD1^A4V^ (Fig 6c and 6d).

### PB treatment reduces the formation of toxic trimer species in de-metallated SOD1^WT^ and an ALS disease mutant, SOD1^A4V^

**Fig 7:**
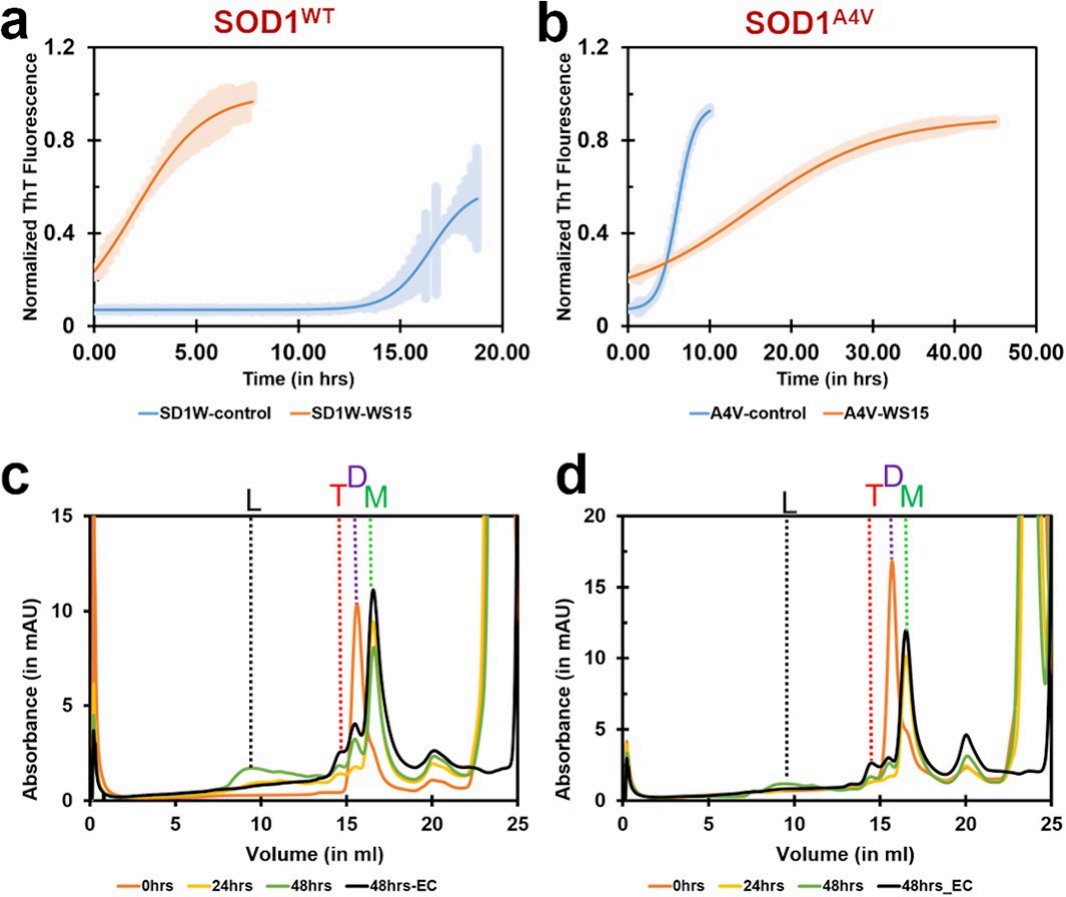
Aggregation studies of de-metallated and reduced SOD1^WT^ and SOD1^A4V^ on treatment with PB. ThT fluorescence was monitored during the co-incubation of de-metallated SOD1^WT^ (50 μM) **(a)** or SOD1^A4V^ (50 μM) (**b**) with PB at 1:30 molar ratio at 37 °C with continuous shaking, under reduced conditions. The values were normalized to the maximal ThT intensity elicited by SOD1^WT^ or SOD1^A4V^ and fitted to a Boltzmann sigmoidal equation. The control and PB-treated species were compared to analyse PB’s effects on the aggregation of SOD1^WT^ and SOD1^A4V^. Analytical SEC of de-metallated SOD1^WT^ (50 μM) **(a)** and SOD1^A4V^ (50 μM) **(b)** with PB were analysed under reducing conditions before and after 24 hrs and 48 hrs incubation at 37ºC. The first peak eluting at the volume was denoted as oligomerized SOD1^WT^ (L) runs at 9.2 ml. The second, third, and fourth peaks were calculated as trimer (T), dimer (D), and monomer (M) species of SOD1^WT^/SOD1^A4V^ running at 14.5 ml, 15.7 ml, and 16.5 ml, respectively. The size-exclusion chromatogram shows how PB binding to SOD1^WT^ promotes shifting towards the formation of large aggregates (L) rather than toxic trimer (T) formation. In the case of SOD1^A4V^, PB eliminates the formation of large aggregates compared to the control (48hrs-EC, EC represents control with EDTA). The monomer (M), dimer (D), trimer (T), and large aggregate (L) species are shown as green, purple, red, and black dotted lines, respectively. All the experiments were performed for n = 3 biological replicates.

We compared the aggregation kinetics of de-metallated SOD1^WT^ and its ALS-causing mutant, SOD1^A4V^, using ThT fluorescence (Fig 7a and 7b). We observed that although de-metallated SOD1^A4V^ exhibited later and slower aggregation kinetics in the presence of PB with an increase in the lag time duration, de-metallated SOD1^WT^, in contrast, exhibited faster and higher rates of ThT signal (Table 3). The ThT-based aggregation assays were also performed for PB site-specific mutants (SOD1^A123F^, SOD1^K9F^, SOD1^L42R^, and SOD1^G10P^). Similar to the metalled-SOD1^WT^ and SOD1^A4V^ conditions, the aggregation analysis is also inconclusive as these mutants, which correspond to the ligand binding residues, cause a loss of protein-ligand interactions (S4b, S4d, S4f and S4h Fig).

The analytical size-exclusion chromatography-based aggregation assays were performed to test the treatment of PB on the de-metallated forms of SOD1^WT^ and SOD1^A4V^. Similar to the metallated aggregation conditions described earlier, PB was also observed to induce the formation of higher aggregate species for SOD1^WT^ at 48hrs (Fig 7c). The formation of trimer species was insignificantly reduced compared to the control. Although the dimer form of SOD1^WT^ completely destabilizes into its monomer form within the first 24 hrs, we observed a reversion of the monomer species into the dimer after 24 hrs. Interestingly, the magnitude of large aggregate species formed in de-metallated SOD1^WT^ is much lesser than its metallated counterpart, all in the presence of PB.

Investigation of the anti-aggregation propensity of PB against SOD1^A4V^ shows that in comparison to the control, treatment with PB reduces the trimer population and exhibits the development of higher aggregates. However, there is no change in the monomeric population at 30X concentration [22] (Fig 7d). The analysis of de-metallated SOD1^A4V^ aggregation in the presence of 60X PB reveals an increase in the aggregated form (L) with a subsequent decrease in the monomer population (S3b Fig) compared to the 30X run (Fig 7d). The formation of dimer and trimer populations is significantly unaltered. This increase in large aggregates reveals that PB directs the formation of large aggregates from monomeric species and does not allow toxic-trimer intermediate formation.

## Discussion

Pathogenic mutations on the SOD1 were shown to destabilize the SOD1 structure through dimer destabilization, metal loss, and disulfide bond disruptions. The essential pathway to SOD1 maturation involves the incorporation of zinc ion into the SOD1 structure, SOD1 dimer formation, and hCCS (a copper chaperone) assisted incorporation of copper ion and intra-disulfide formation into the SOD1 structure [37,38].

As described previously, pathological SOD1 mutations in ALS have a toxic gain of function, destabilizing the native homodimer structure and forming aggregates through a non-native trimer intermediate. In the analytical SEC-based aggregation assays, the proposed molecule shows a significant reduction in the trimerization of SOD1 in both wild-type and disease mutant with an increase in the dimer population and larger aggregate forms. Large aggregates have been shown to play a protective role and are not responsible for neurodegeneration [12,13,39]. The proposed molecule’s ability to reduce the lethal SOD1 trimers can be exploited against neurodegeneration in ALS to serve as a potential therapeutic candidate.

The reported compounds bind to the single surface-exposed tryptophan residue, W32 [20–22], or at the free cysteine residue, C111, at the dimer interface [23,25,26,30]. These compounds target through the camouflage of the former residue from tryptophan oxidation or through dimer stabilization by π–π stacking covalently linked molecules such as ebselen, ebsulphur, and other derivatives at the C111 residues. Aggregate conformational analysis of all aggregate formations is lacking and required for a stoic comparison of PB with these previously published compounds.

Our study revealed that a novel linear chain lipidic compound, PB, has the potential to bind to SOD1 at two new sites, which are different from the ones already discovered previously. One of these sites lies at the dimer interface, close to the intra-disulfide bond formed by the residues C57 and C146. The other site of PB binding is found at the lateral region of SOD1 near the electrostatic loop. On careful inspection of the SOD1–Phialomustin-B crystal complex, the compound lies close to the intra-disulfide bond formed by C57 and C146 at the dimer interface. Also, it lies close to the nearby binding site residue N53. This residue has been shown to destabilize the trimer populations on a mutation to N53I [11]. We speculate that the presence of a linear lipophilic moiety of PB exerts similar trimer destabilizing effects on SOD1 as occurred in the N53I mutant. Although ebselen was found to chemically induce the intra-disulfide formation through an intermediate selenylsulphide bond under a reducing environment [25], we speculate that our compound, PB, can protect the SOD1 dimer interface by zip-locking through structural, non-covalent interactions under similar reducing conditions.

In addition, PB also binds near the electrostatic loop. At this second binding site, it interacts with some critical residues like N86 and D124. SOD1^N86S^ and SOD1^N86K^ cause pathogenic ALS wherein the former mutant advances to as aggressive ALS as SOD1^A4V^ [40,41]. We hypothesize that PB might be capable of protecting the mutation-related structural changes by stabilizing the local conformation. Additionally, D124 is an important mutant for metal binding as it directly links the copper and zinc-binding sites [42,43]. Hence, we speculate that PB has the potential to stabilize the local structural conformation by engaging the intermolecular interactions and preventing any other structural changes. The compound behaves well on further biophysical assays to see the effects of PB on the aggregation of SOD1 and its mutants. Biochemical studies validated the ligand, PB, binding to SOD1. SOD1 was found to have a substantial binding affinity to PB with a K_D_ value of 3.7 ± 1.2 μM. Binding affinities were also determined for all four mutants as described previously. The increase in the K_d_ values indicates weak to no binding of PB to these site-specific mutants suggesting that these residues are important for the PB interaction with SOD1.

Previous studies like Ray et al. [44] delved into the screening of multiple compounds against SOD1 wild-type and mutants using the aggregation assay based on the calculation of dimer reduction. But, it lacks structural evidence that the selected compounds bind to the SOD1 dimer interface as predicted through the *in-silico* studies [44]. Previous studies have revealed that the formation of trimer species in SOD1 causes the fatal form of aggregation instead of the aggregation involving non-trimer aggregates [11–13]. For this reason, the trimer species are stated to be toxic. Additionally, the SOD1^A4V^ species are faster in aggregation due to variations in the trimer species compared to the SOD1^WT^ species [35].

Our studies on testing the aggregation propensity of PB on SOD1^WT^ and SOD1^A4V^ showed a correlation in both the ThT and the analytical size-exclusion chromatography assays. Under metallated conditions, the ThT aggregation studies revealed earlier but slower aggregate formation in treating PB on SOD1^WT^ and SOD1^A4V^. In the case of SOD1^WT^, we speculate the formation of large aggregates in the presence of lower populations of the trimer species, as seen in the analytical size exclusion chromatography. This justifies the delayed elongation on the treatment of PB compared to the sharp elongation of control SOD1^WT^. On the contrary, it is observed that treating PB on SOD1^A4V^ abolishes the large aggregate formation but also reduces the trimer populations drastically. This variation justifies the significant reduction in the ThT signal intensity on treatment with PB compared to control SOD1^A4V^. The size-exclusion chromatography based aggregation results are in accordance with the previously reported results [12,45,46], wherein it has been observed that the formation of soluble and specifically large aggregates is not lethal to the viability of motor neurons in cells unless there are other factors, such as insoluble aggregates involved contributing to the cytotoxicity of the mutant SOD1. The formation of the oligomeric population reveals that PB might induce a similar cytoprotective effect, as described in the previous studies. Previous reports suggest that trimer destabilization plays a potential therapeutic strategy in designing SOD1 anti-aggregator molecules.

EDTA was used to chelate the zinc ions from the SOD1 structure for the de-metallated variation. The aggregation kinetics of de-metallated SOD1^WT^ and SOD1^A4V^ in the presence of PB are different from their metallated counterparts. The ThT studies revealed that although PB seems effective for SOD1^A4V^, it does not seem to improve the aggregation of SOD1^WT^. Detailed size-exclusion chromatography-based investigations revealed that metal chelation changes the way aggregation happens in SOD1 compared to its metallated states. In de-metallated conditions, both SOD1^WT^ and SOD1^A4V^ showed a major monomerization step within the first 24 hours. Although control kinetics do not form large aggregates from the SOD1^WT^ monomers, treatment with PB induces the formation of large aggregates and a surprisingly additional dimerization step. SOD1^A4V^, on the other hand, does not cause the dimerization step. Still, it induces higher aggregate species like SOD1^WT^ with a reduction in a monomeric population at a higher concentration of PB (60X), stating the formation of oligomers without intermediate toxic-trimer formation. The size-exclusion chromatography results of PB with aggregated forms of SOD1^WT^ and SOD1^A4V^ revealed better inhibition of dimer formation than other molecules like isoproterenol and 5-FU. However, the latter was later discovered to bind at the tryptophan W32 site of the SOD1 [22].

## Conclusion

One of the primary objectives of ALS research is to evolve novel therapeutic drugs for effective treatment. Only two prescription drugs exist to treat ALS–Riluzole and Edaravone [15,47]. The approved drugs for ALS to date have only moderate efficacy in treating the disease. There are no reports about the known drugs to inhibit SOD1 toxic trimerization. Drug discovery in ALS focuses on compounds ranging from aromatic drug-like molecules targeting the W32 site to covalently binding molecules targeting the free cysteine C111 to zip-lock the dimer interface. The identified novel lipidic molecule, PB, binds to the dimer interface of SOD1 close to the intra-disulfide region and a lateral site. It is the first non-covalent small molecule that binds at the dimer interface of SOD1, close to the intra-disulfide bond. PB has a promising binding affinity to SOD1, significantly reducing toxic trimer species by increasing dimer stability and converting monomeric species into large soluble, potentially non-lethal aggregates. Thus, Phialomustin-B, a fungal derivative, is a promising candidate for further developing a ligand library with better affinity and anti-aggregation propensity towards therapeutic potential in ALS.

## Materials and methods

### Phialomustin-B isolation

The compound, Phialomustin-B was obtained as described previously^23^. Briefly, the endophytic fungus, *Phialophora mustea* was extracted from the internal tissues of the Corms of *Crocus sativus* and grown on potato dextrose agar (Difco) in pure culture. 25 litres culture broth of *Phialophora mustea* was subjected to extraction with ethyl acetate and subsequent concentration under reduced pressure. The concentrated extract was subjected to column chromatography over silica gel (230–400 mesh) using a gradient of hexane and ethyl acetate (from 100% hexane to 100% ethyl acetate). Separation by repeated column chromatography over silica gel (230–400 mesh) with a step gradient of hexane and ethyl acetate (from 30% ethyl acetate to 100% ethyl acetate) led to the isolation of a compound designated as *Phialomustin-B* (PB).

### Cloning, expression and purification of SOD1^WT^ and SOD1^A4V^

The full-length constructs of SOD1-wild type (aa: 1–145) and its corresponding SOD1 disease mutant, SOD1^A4V^, were obtained for protein production and biochemical studies. The cloning and expression of plasmid (pET-M11) containing the protein of interest has been already described [21] The plasmid with the positive clone was transformed into *E*.*coli* BL21 DE3 (Stratagene) cells [20,21]. These cells were grown at 37°C till the culture reached OD_600_ = 0.6. For the disease mutants, the SOD1 expression was induced in the presence of 0.5 M IPTG at 18° C. The cells were harvested by centrifugation of the culture at 7000 rpm. The cells were suspended in a lysis buffer containing 30mM KH_2_PO_4_, 300mM NaCl, 5mM β-mercaptoethanol, 1mM PMSF, lysozyme, DNase, and complete protease inhibitor (CPI) cocktail. The suspended cells were subjected to sonication (Vibra Cell) at 30% amplitude with a 3-second pulse and 5-second rest. The supernatant was loaded onto a Ni-NTA column (HisTrap FF, GE Healthcare, USA) for metal affinity purification and eluted with 200 mM of Imidazole. The His-tag was removed by TEV protease by incubating the eluted samples at 4°C on a rocker overnight. Subsequently, it was concentrated and separated from remaining impurities by size exclusion chromatography (Superdex 75G 16/60 from GE Healthcare, USA) on an AKTA Pure (GE Healthcare, USA).

### Cloning, expression, and purification of SOD1-PB site-specific mutants

A gene for SOD1 was cloned into the pETM11 vector (6.02Kb + 0.45bp). The polymerase chain reaction was performed with this construct as a template to generate SOD1 mutants K9F, G10P, L42R, and A123F using specific primers depicted in Table 4. Phusion HF^®^ polymerase from NEB (New England Biolabs Inc., Ipswich, MA, USA) and dNTP mix were used for the mutagenesis. The amplified constructs were subjected to *DpnI* (TaKaRa Bio Inc.) treatment to separate mutated constructs from template DNA and transformed them into DH5ɑ. Confirmation of mutants was done using Sanger sequencing of the plasmids isolated from selective colonies. The confirmed mutant plasmids were again transformed into BL21-DE3 hosts. These mutants were induced with IPTG and optimal temperature conditions and overexpressed in the *E*. *coli* host. The purification of mutant proteins was carried out as described for the SOD1^WT^ protein.

**Table 4:** Details of the forward and reverse primers used to perform site-directed mutagenesis to generate SOD1-PB site-specific mutants.

Mutant	Forward Primer	Reverse Primer
A123F	GTG GTC CAT GAA AAA AGA GAT GAC TTG GGC AAA	TTT GCC CAA GTC ATC TCT TTT TTC ATG GAC CAC
L42R	GGA CTG ACT GAA GGC CGG CAT GGA TTC CAT GTT	AAC ATG GAA TCC ATG CCG GCC TTC AGT CAG TCC
K9F	GCC GTG TGC GTG CTG TTT GGC GAC GGC CCA GTG	CAC TGG GCC GTC GCC AAA CAG CAC GCA CAC GGC
G10P	GTG TGC GTG CTG AAG CCC GAC GGC CCA GTG CAG	CTG CAC TGG GCC GTC GGG CTT CAG CAC GCA CAC

### Crystallization, data collection, and structural determination

The apo crystals of SOD1^WT^ (8–9 mg/ml in 20 mM Tris pH 8.0) were obtained in the crystallization conditions containing 1.2–1.4 M sodium citrate (pH = 6.5). The protein-ligand ratios of 1:5, 1:10, and 1:20 were set for the co-crystallization studies. Drops of 2 μl protein-ligand complex mixed with 2 μl reservoir solution in 24-well plates were equilibrated against a 500 μl reservoir solution. The cryoprotectant, 5% glycerol, was added during crystal harvesting.

The diffraction quality of the crystals was checked on the in-house X-Ray diffractometer (Rigaku XtaLAB Synergy-S) integrated with a Kappa goniometer and HyPix 6000HE detector. A high-resolution X-ray crystallographic data was collected on the beamline BL-14 at ESRF, Grenoble, France. The diffraction data were merged, indexed, integrated, and scaled using HKL-3000 v.721.3 [48]. The molecular replacement method was performed using Phaser-MR [49] using a search model of the SOD1 dimer (PDB ID– 5YTO). The structure solution yielded five dimers of SOD1 in the asymmetric unit. At four sites, an apparent electron density for the PB ligand was observed in the 2m|Fo|—D|Fc| and the Polder difference Fourier maps [50]. The PB ligand’s energy minimized coordinates and crystal information file (CIF) were obtained using the grade web server (http://grade.globalphasing.org).

The crystal structure of the SOD1-PB complex was refined by using module *phenix*.*refine* of the *PHENIX* package [51]. Subsequent refinement cycles and model-building rounds using Coot [52] were carried out to obtain the final structure. The final refinement yielded the real-space correlation coefficient (RSCC) value of ~0.73 for the PB molecules in the structure of the complex, confirming the ligand binding. The X-ray data collection, scaling, and refinement statistics are summarized in Table 1. The final structures and refined maps were visualized and illustrated using PyMOL v.1.3r [53]. The MOLPROBITY program [54] was used to assess the stereochemistry of these crystal structures. The structure factors and structural coordinates of the SOD1-PB complex have been deposited in the RCSB (PDB ID: 7XX3).

### Binding analysis by microscale thermophoresis (MST) assay

Binding studies were performed for SOD1^WT^ and the ligands using microscale thermophoresis (MST). The purified recombinant SOD1^WT^ protein was labeled at its cysteine residues using RED- Maleimide dye (NanoTemper Technologies GmbH, Munich, Germany. The protein buffer system was exchanged for the RED-Maleimide labeling buffer. 10 μM of SOD1 was mixed with 20 μM of RED-Maleimide dye and incubated in the dark for 30 minutes. The labeled protein was eluted with PBS buffer using a PD-10 desalting column post-incubation. The collected elutes were tested for fluorescence intensity. The fluorescent counts evaluated the protein labeling at 20–30% of LED excitation and medium MST power. MO.Control 1.5.3 and MO.Affinity Analysis 2.3 softwares were used to collect and analyze the MST data, respectively. Like the SOD1^WT^, the disease mutant SOD1^A4V^ and other binding site mutants SOD1^K9F^, SOD1^G10P^, SOD1^L42R^, and SOD1^A123F^ were also successfully labeled. The labeled protein was titrated against an unlabelled ligand for 16 dilutions, with concentrations starting from 1 mM. The thermophoretic curves were used to determine a K_d_ value. The experiments were carried out in triplicates. The data for the binding of compounds and subsequent calculation of K_d_ value was carried out by plotting the fraction bound data points against the ligand concentrations (in log scale) as used in the experiment. The sigmoidal curve generated with the above data was used to determine the K_d_ value. The figures were prepared using GraphPad Prism 8.4.3 (GraphPad, San Diego, California).

### Aggregation analysis by Thioflavin T (ThT) assay

Aggregation assays were performed on SOD1^WT^, the disease mutant A4V, and ligand-binding site-specific mutants to check the anti-aggregation activity of PB on the former. The binding of Thioflavin T (ThT) to the amyloid fibrils of aggregated proteins exhibits a fluorescence intensity used to measure the aggregation kinetics of the proteins [55,56]. The ThT assay for the SOD1 aggregation was carried out per the recently published protocol [23]. 96-well clear-bottomed black (opaque walls) plates (Corning) were used to measure the increase in fluorescence intensity due to the dye’s binding, Thioflavin-T to aggregates SOD1. The protocol for the ThT-based aggregation assay involves 50 μM of SOD1 in incubation in a buffer system containing 1x phosphate-buffered saline (PBS), 10 μM ThT, 20 mM DTT (30 mM for wild-type) in the presence and absence of 5 mM EDTA. The anti-aggregation efficacy of the compounds was tested in the presence of protein in the above buffer. 200 μl of the assay mixture was added to 96-well clear-bottomed opaque-black assay plates, carefully sealed with an adhesive plate sealant film. Bottom optic-based readings are taken for the plate in Tecan Spark (Thermo Fischer). The plate was incubated at 37 °C and shaken at 180 rpm speed in a double-orbital mode for 330 sec. The fluorescence intensity readings were measured every 15 minutes at an excitation wavelength of 440 nm and an emission wavelength of 490 nm. The ligand concentration used during the initial optimization was around 30 times the concentration of protein used. The concentration of the protein and ligand used for the experiment was 50 μM and 1500 μM. Each cycle of the kinetic plate readings was recorded every 15 min, totalling up to 192 cycles for 48 hours. The optimal gain of the fluorescence reading was optimized to 20% of the total gain.

The experiments were performed in triplicate. The normalized readings were averaged and plotted. The average plots were fitted to the Boltzmann sigmoidal equation (Eq 1) [57]. R^2^, the coefficient of determination, was used to cap the quality of the fits to anything more significant than 0.8.

$$F=F_{i}+\frac{F_{f}-F_{i}}{1+e^{\left(\frac{{(t}_{50}-t)}{k}\right)}}$$ (1)

Where F_i_ corresponds to the initial fluorescence value, F_f_ corresponds to the final fluorescence value, t_50_ refers to the time taken to reach half-maximal fluorescence, and k describes the gradient of the curve. The lag values were used to analyse the efficiency of small molecule modulators to alleviate the aggregation of SOD1^WT^ and its disease mutants [58]. The equation for the lag phase calculation is given in Eq 2 where t_lag_ is the lag time, t_50_ is the time to attain half of the maximal fluorescence and k_app_ corresponds to the apparent rate constant.


$$t_{lag}=t_{50}-\frac{2}{k_{app}}$$ (2)

### Aggregation analysis by analytical size exclusion chromatography (SEC) assay

The anti-aggregation activity of PB was validated on the SOD1^WT^ and the disease mutant (SOD1^A4V^) through size exclusion chromatography. The analytical size-exclusion chromatography is a gold standard method for comprehensively evaluating protein species through their molecular weight variations detected by the protein peaks’ elution volume (V_e_). The purified SOD1 proteins in their dimer conformation were tested on the SEC analytical column (Superdex 200 pg 10/300 GL, GE Healthcare). As previously reported protocol [22], 50 μM of the protein was incubated with 1500 μM of PB (30X) in a buffer system containing 20 mM Tris-HCl (pH = 7.4), 150 mM NaCl and 20mM DTT with the addition of 5mM EDTA (for de-metallated conditions) to induce aggregation. The same experiment was performed for PB (60X) for SOD1^A4V^. All the setup mixtures were incubated at 37°C with constant shaking for 48 hours. Negative controls (buffer only; buffer + EDTA; protein only; protein + PB) and positive controls (protein + EDTA) were also set up for the aggregation analysis. Vehicle control (protein + DMSO) was also included during testing to rule out the solvent effects on the aggregation of proteins. The samples were tested at 0, 24, and 48 hours. The chromatograms were analysed based on their elution volumes to determine the extent of ligand-induced alleviation of protein aggregation. Faster elution volumes detected higher molecular weight forms in the sample. The peaks seen in the void volumes were not considered for analysis.

## Supporting information

S1 Fig Crystal structure of human SOD1 complex with an unknown ligand. The surface/cartoon model of the homodimer assembly of SOD1-UNK shows binding from an unknown linear molecule, UNK (shown in the pink sticks), at the SOD1 dimer interface. The molecule lies very close to the intra-disulfide bond formed by the residues C57 and C146. The 2mFo–DFc map is contoured at 1σ level and shown as blue mesh.
(DOCX)

S2 Fig Aggregation studies of de-metallated and reduced SOD1^WT^ and SOD1^A4V^ on treatment with PB. Analytical SEC of metallated SOD1^WT^ (a), de-metallated SOD1^WT^ (b), metallated SOD1^A4V^ (c) and de-metallated SOD1^A4V^ (d) in the absence of PB were analyzed under reducing conditions before and after 24 hrs and 48 hrs incubation at 37ºC. The monomer (M), dimer (D), trimer (T), and large aggregate (L) species are shown as green, purple, red, and black dotted lines, respectively. All the experiments were performed for n = 3 biological replicates.
(DOCX)

S3 Fig Aggregation studies of de-metallated and metallated SOD1^A4V^ on treatment with 60x PB. Analytical SEC of metallated SOD1^A4V^ (a) and de-metallated SOD1^A4V^ (b) in the absence of PB were analyzed under reducing conditions before and after 24 hrs and 48 hrs incubation at 37ºC. The monomer (M), dimer (D), trimer (T), and large aggregate (L) species are shown as green, purple, red, and black dotted lines, respectively. All the experiments were performed for n = 3 biological replicates.
(DOCX)

S4 Fig Aggregation studies of de-metallated and reduced SOD1-PB site specific mutants on treatment with PB. ThT fluorescence was monitored during the co-incubation of **(a)** SOD1^A123F^ (50 μM) with PB at 1:30 molar ratio (b) in the presence of 5mM EDTA, (c) SOD1^K9F^ (50 μM) with PB at 1:30 molar ratio (d) in the presence of 5mM EDTA (e) SOD1^G10P^ (50 μM) with PB at 1:30 molar ratio (f) in the presence of 5mM EDTA (g) SOD1^A123F^ (50 μM) with PB at 1:30 molar ratio (h) in the presence of 5mM EDTA, at 37 °C with continuous shaking, under reduced conditions. The values were normalized to the maximal ThT intensity and fitted to a Boltzmann sigmoidal equation. The control and PB-treated species were compared to analyse PB’s effects on the aggregation of SOD1 and SOD1.. All the experiments were performed for n = 3 biological replicates.
(DOCX)
