# Supplementary material for: Structural insights into the modulation Of SOD1 aggregation By a fungal metabolite Phialomustin-B: Therapeutic potential in ALS
Source: PLoS One. 2024 Mar 6;19(3):e0298196. doi: 10.1371/journal.pone.0298196 (PMC10917278; doi:10.1371/journal.pone.0298196)
Supplement: S1 Fig — The surface/cartoon model of the homodimer assembly of SOD1-UNK shows binding from an unknown linear molecule, UNK (shown in the pink sticks), at the SOD1 dimer interface. The molecule lies very close to the intra-disulfide bond formed by the residues C57 and C146. The 2mFo–DFc map is contoured at 1σ level and shown as blue mesh. (DOCX) [file pone.0298196.s001.docx]

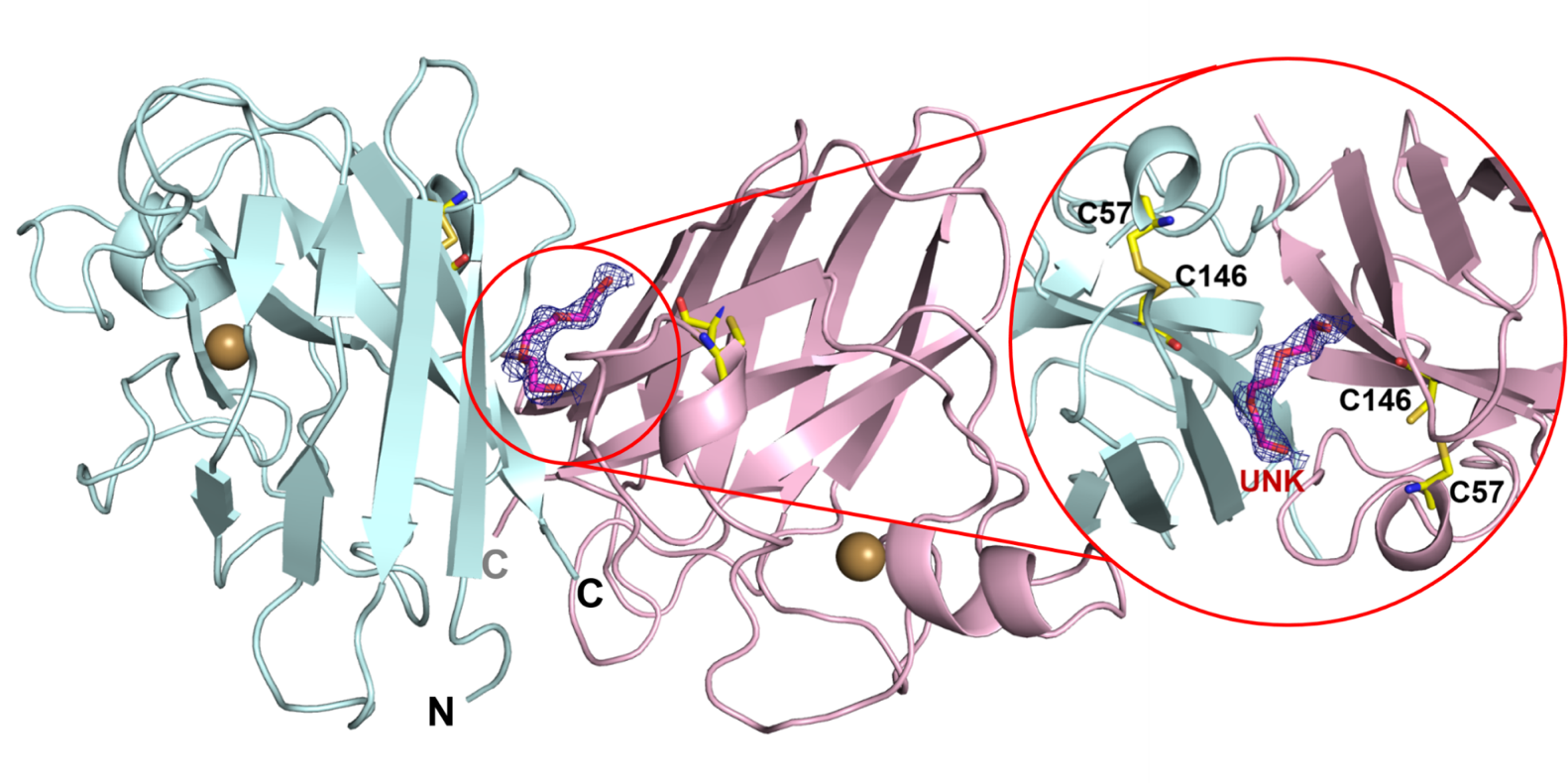


Figure S1. Crystal structure of human SOD1 complex with an unknown ligand. The surface/cartoon model of the homodimer assembly of SOD1-UNK shows binding from an unknown linear molecule, UNK (shown in the pink sticks), at the SOD1 dimer interface. The molecule lies very close to the intra-disulfide bond formed by the residues C57 and C146. The 2mFo – DFc map is contoured at 1σ level and shown as blue mesh.
