# Supplementary material for: Structural insights into the modulation Of SOD1 aggregation By a fungal metabolite Phialomustin-B: Therapeutic potential in ALS
Source: PLoS One. 2024 Mar 6;19(3):e0298196. doi: 10.1371/journal.pone.0298196 (PMC10917278; doi:10.1371/journal.pone.0298196)
Supplement: S3 Fig — Analytical SEC of metallated SOD1A4V (a) and de-metallated SOD1A4V (b) in the absence of PB were analyzed under reducing conditions before and after 24 hrs and 48 hrs incubation at 37ºC. The monomer (M), dimer (D), trimer (T), and large aggregate (L) species are shown as green, purple, red, and black dotted lines, respectively. All the experiments were performed for n = 3 biological replicates. (DOCX) [file pone.0298196.s003.docx]

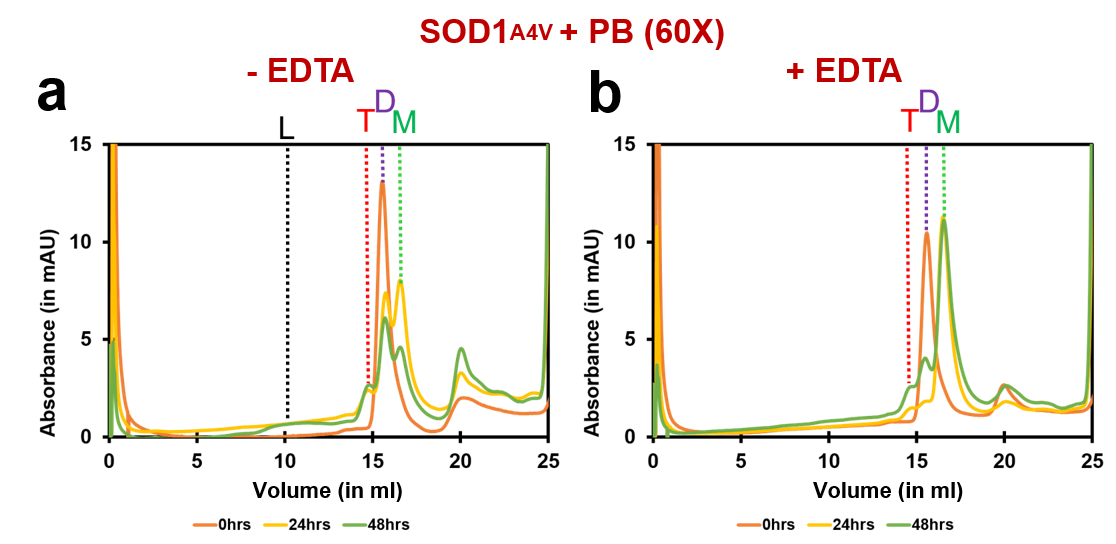


**Figure S3.** Aggregation studies of de-metallated and metallated SOD1^A4V^ on treatment with 60x PB. Analytical SEC of metallated SOD1^A4V^ (a) and de-metallated SOD1^A4V^ (b) in the absence of PB were analyzed under reducing conditions before and after 24 hrs and 48 hrs incubation at 37ºC. The monomer (M), dimer (D), trimer (T), and large aggregate (L) species are shown as green, purple, red, and black dotted lines, respectively. All the experiments were performed for n = 3 biological replicates.
