# Supplementary material for: Structural insights into the modulation Of SOD1 aggregation By a fungal metabolite Phialomustin-B: Therapeutic potential in ALS
Source: PLoS One. 2024 Mar 6;19(3):e0298196. doi: 10.1371/journal.pone.0298196 (PMC10917278; doi:10.1371/journal.pone.0298196)
Supplement: S4 Fig — ThT fluorescence was monitored during the co-incubation of (a) SOD1A123F (50 μM) with PB at 1:30 molar ratio (b) in the presence of 5mM EDTA, (c) SOD1K9F (50 μM) with PB at 1:30 molar ratio (d) in the presence of 5mM EDTA (e) SOD1G10P (50 μM) with PB at 1:30 molar ratio (f) in the presence of 5mM EDTA (g) SOD1A123F (50 μM) with PB at 1:30 molar ratio (h) in the presence of 5mM EDTA, at 37 °C with continuous shaking, under reduced conditions. The values were normalized to the maximal ThT intensity and fitted to a Boltzmann sigmoidal equation. The control and PB-treated species were compared to analyse PB’s effects on the aggregation of SOD1 and SOD1.. All the experiments were performed for n = 3 biological replicates. (DOCX) [file pone.0298196.s004.docx]

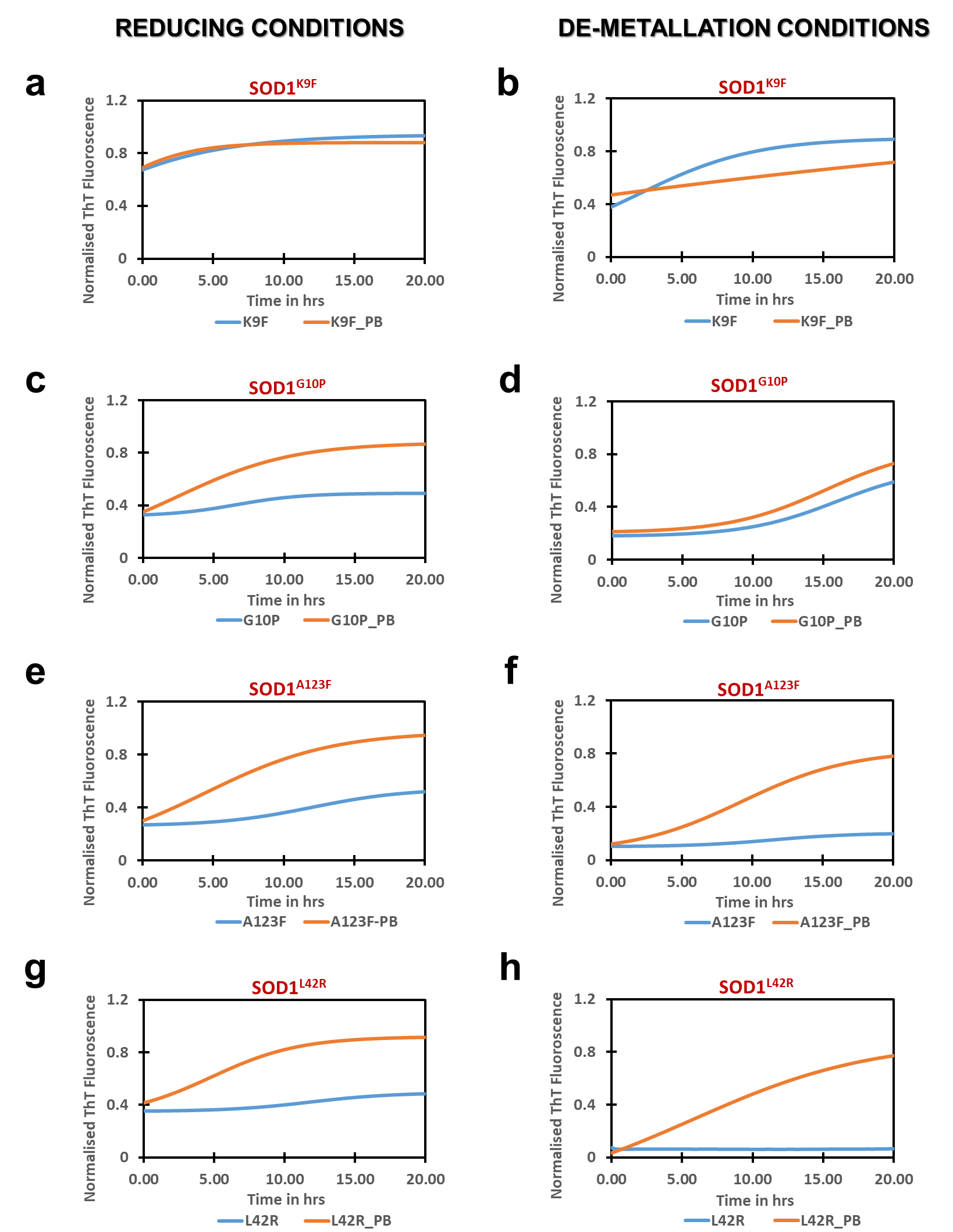


**Figure S4**. Aggregation studies of de-metallated and reduced SOD1 mutants on treatment with PB. ThT fluorescence was monitored during the co-incubation of (a) SOD1A123F (50 µM) with PB at 1:30 molar ratio (b) in the presence of 5mM EDTA, (c) SOD1K9F (50 µM) with PB at 1:30 molar ratio (d) in the presence of 5mM EDTA (e) SOD1G10P (50 µM) with PB at 1:30 molar ratio (f) in the presence of 5mM EDTA (g) SOD1A123F (50 µM) with PB at 1:30 molar ratio (h) in the presence of 5mM EDTA, at 37 °C with continuous shaking, under reduced conditions. The values were normalized to the maximal ThT intensity and fitted to a Boltzmann sigmoidal equation. The control and PB-treated species were compared to analyse PB's effects on the aggregation of SOD1 and SOD1. All the experiments were performed for n = 3 biological replicates.
